# *Ralstonia solanacearum* fatty acid composition is determined by interaction of two 3-ketoacyl-acyl carrier protein reductases encoded on separate replicons

**DOI:** 10.1186/s12866-015-0554-x

**Published:** 2015-10-22

**Authors:** Sai-Xiang Feng, Jin-Cheng Ma, Ji Yang, Zhe Hu, Lei Zhu, Hong-Kai Bi, Yi-Rong Sun, Hai-Hong Wang

**Affiliations:** Guangdong Provincial Key Laboratory of Protein Function and Regulation in Agricultural Organisms, College of Life Sciences, South China Agricultural University, No.483 Wushan Road, Tianhe, Guangzhou, 510642 P. R. China; Departments of Microbiology and Biochemistry, University of Illinois at Urbana-Champaign, Urbana, IL 61801 USA; Department of Pathogenic Biology, Jiangsu Key Laboratory of Pathogenic Biology, Nanjing Medical University, Nanjing, Jiangsu 210029 China; Guangzhou Institutes of Biomedicine and Health, Chinese Academy of Sciences, Guangzhou, Guangdong 510530 China

**Keywords:** *R. solanacearum*, 3-ketoacyl-ACP reductase, Type II fatty acid synthase system

## Abstract

**Background:**

FabG is the only known enzyme that catalyzes reduction of the 3-ketoacyl-ACP intermediates of bacterial fatty acid synthetic pathways. However, there are two *Ralstonia solanacearum* genes, RSc1052 (*fabG1*) and RSp0359 (*fabG2*), annotated as encoding putative 3-ketoacyl-ACP reductases. Both FabG homologues possess the conserved catalytic triad and the N-terminal cofactor binding sequence of the short chain dehydrogenase/reductase (SDR) family. Thus, it seems reasonable to hypothesize that *RsfabG1* and *RsfabG2* both encode functional 3-ketoacyl-ACP reductases and play important roles in *R. solanacearum* fatty acid synthesis and growth.

**Methods:**

Complementation of* Escherichia colifabG* temperature-sensitive mutant with *R. solanacearum fabGs* encoded plasmids was carried out to test the function of *RsfabGs* in fatty acid biosynthesis. RsFabGs proteins were purified by nickel chelate chromatography and fatty acid biosynthetic reaction was reconstituted to investigate the 3-ketoacyl-ACP reductase activity of RsFabGs* in vitro*. Disruption of both *RsfabG* genes was done via DNA homologous recombination to test the function of both *RsfabG in vivo*. And more we also carried out pathogenicity tests on tomato plants using RsfabG mutant strains.

**Results:**

We report that expression of either of the two proteins (RsFabG1 and RsFabG2) restores growth of the *E. coli* fabG temperature-sensitive mutant CL104 under non-permissive conditions. *In vitro* assays demonstrate that both proteins restore fatty acid synthetic ability to extracts of the *E. coli* strain. The *RsfabG1* gene carried on the *R. solanacearum* chromosome is essential for growth of the bacterium, as is the case for *fabG* in *E. coli*. In contrast, the null mutant strain with the megaplasmid-encoded *RsfabG2* gene is viable but has a fatty acid composition that differs significantly from that of the wild type strain. Our study also shows that RsFabG2 plays a role in adaptation to high salt concentration and low pH, and in pathogenesis of disease in tomato plants.

**Conclusion:**

*R. solanacearum* encodes two 3-ketoacyl-ACP reductases that both have functions in fatty acid synthesis. We supply the first evidence that, like other enzymes in the bacterial fatty acid biosynthetic pathway, one bacterium may simultaneously possess two or more 3-oxoacyl-ACP reductase isozymes.

**Electronic supplementary material:**

The online version of this article (doi:10.1186/s12866-015-0554-x) contains supplementary material, which is available to authorized users.

## Background

Fatty acid biosynthesis is essential for the survival of mammals, plants, fungi and bacteria [1, 2]. Using acetyl-CoA and malonyl-CoA as the initiating substrate and building block, respectively, these organisms share a common set of biochemical reactions to extend fatty acyl chains by two carbon atoms per cycle [1–3]. In most bacteria and plants, fatty acids are synthesized by a discrete and highly conserved group of enzymes designated the Type II or dissociated fatty acid synthase (FAS) system [2–5]. As a key feature of the FAS II system, the hydrophobic fatty acyl intermediates are shuttled from enzyme to enzyme by a small, highly acidic acyl carrier protein (ACP) [3, 4]. To date, the FAS II system has been most extensively studied in the *Escherichia coli* model system, in which all the enzymes required for fatty acid synthesis have been identified and characterized biochemically [2–4].

The genes encoding fatty acid synthetic enzymes are highly conserved in bacteria, and in many cases, their genomic arrangement is also conserved [2, 3, 6]. Although the basic steps in the fatty acid synthesis cycle are common to all bacteria [3, 4], abundant exceptions to the *E. coli* paradigm are present in other bacteria [4, 7, 8]. In general, a discrete enzyme encoded by a single chromosomal locus catalyzes each step of the elongation pathway. However, although some bacteria encode isozymes, in most cases these enzymes have differing specificities [7, 8]. One example is *Enterococcus faecalis*, which encodes two homologues each of FabZ and FabF. However, this bacterium uses one of the FabZ homologues (now called FabN) and one of the FabF homologues (now called FabO) to perform the unsaturated fatty acid synthetic functions performed by *E. coli* FabA and FabB [9, 10].

Another example is the enzyme enoyl-ACP reductase, which catalyzes the NAD (P) H-dependent reduction of the enoyl-ACP double bond in the last step of the elongation cycle [7]. Several bacteria have two enoyl-ACP reductases that can be either of the same or of different protein families [11–13]. In some cases one of the enzymes is responsible for supporting a fatty acid synthetic rate that allows wild type growth. The rationale for this duplication of enzyme activity is unknown, but the two enzymes of a given bacterium often differ in their inhibition by triclosan, a man-made biocide [11–14].

**Fig. 1:**
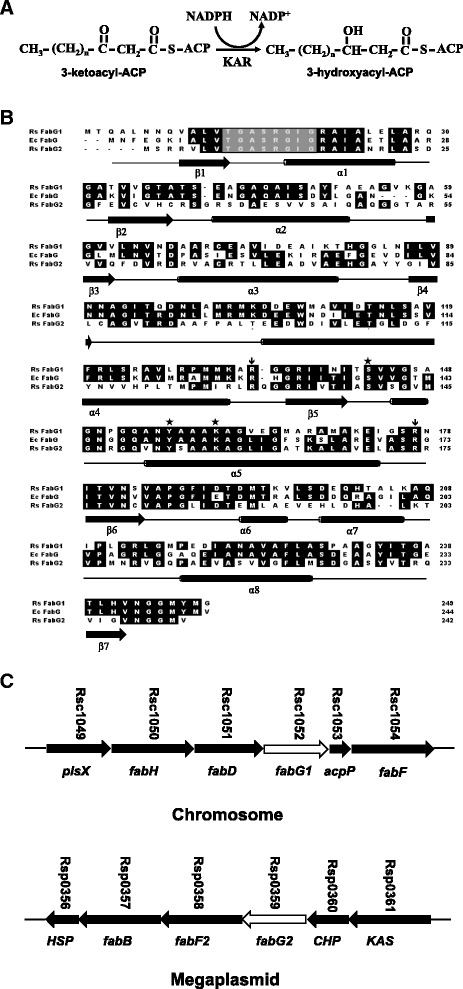
The 3-ketoacyl-ACP reductase (KAR) reaction, alignment of *R. solanacearum* FabG1 and FabG2 with *E. coli* FabG and organization of the *R. solanacearum* fatty acid biosynthesis gene clusters. Panel **a**, The KAR reaction. Panel **b**, Alignments of *R. solanacearum* FabG1 and FabG2 with *E. coli* FabG. Rs and Ec denote *R. solanacearum* and *E. coli*, respectively. The cofactor binding sequence (Gly motif [GlyXXXGlyXGly]) is boxed. The two arginine residues thought to bind the ACP moiety are highlighted by arrows and the catalytic triad residues are highlighted by asterisks. The alignment was done with Clustal W based on identical residues. Panel **c**, Organization of the *R. solanacearum* fatty acid biosynthesis gene clusters. The thick arrows indicate the relative sizes of the genes. The numbers above the arrows indicate the gene designations in the *R. solanacearum* of Comprehensive Microbial Resource (CMR) database, and the gene names below the arrows indicate the *E. coli* genes that correspond to the open reading frames in the *R. solanacearum* cluster. HSP indicates hypothetical signal peptide protein. CHP indicates conserved hypothetical protein. KAS indicates putative 3-ketoacyl-acyl carrier protein synthase

In the bacterial fatty acid synthesis pathways studied to date, only a single enzyme, FabG, has been found to catalyze the reduction of 3-ketoacyl-ACPs to 3-hydroxy acyl-ACPs [2, 3] (Fig. 1a). FabG proteins are particularly difficult to annotate because they are members of the short-chain dehydrogenase/reductase (SDR) protein superfamily, which constitutes one of the largest protein superfamilies, with many bacterial members [15, 16] (Fig. 1b). Hence, annotation of a gene as encoding a 3-ketoacyl-ACP reductase is much more likely to be accurate if the gene is located within a cluster of genes that are good candidates for having roles in fatty acid synthesis. That said, our prior work showed that only one of the two *Lactococcus lactis* annotated *fabG* genes was involved in fatty acid synthesis, although both genes had plausible genome contexts [17].

We report the first example of a bacterium that encodes two functional FabG homologues. This bacterium is *Ralstonia solanacearum*, a soil-borne, destructive plant pathogen that has a global distribution and an unusually wide host range [18]. Functional characterization of the two proteins shows that they both are active in fatty acid synthesis but play different roles in determining the cellular fatty acid profile, the response to environmental stress, and pathogenesis.

## Results

### Two *R. solanacearum* genes annotated as *fabG* homologues

Two *R. solanacearum* genes, called *fabG1* and *fabG2* (*RsfabG1* and *RsfabG2* in this study), were annotated as encoding homologues of *E. coli* FabG, the essential 3-ketoacyl-ACP reductase [18]. The *RsfabG1* gene (RSc1052) is located in a chromosomal cluster of putative fatty acid synthesis genes (*fabH*, *fabD*, *acpP* and *fabF*), based on alignments of their gene products with the *E. coli* proteins, whereas the *RsfabG2* gene (RSp0359) lies within a second putative fatty acid synthesis gene cluster (*fabB*, *fabF2* and *KAS*) located on the megaplasmid (Fig. 1c). Sequence alignments indicated that RsFabG1 and RsFabG2 are 65 % and 43 % identical to *E. coli* FabG, respectively, and showed that the catalytically active short chain dehydrogenase/reductase (SDR) family triad (Ser, Tyr and Lys) and the N-terminal cofactor binding sequence (Gly motif [GlyXXXGlyXGly]) defined by the X-ray crystal structures of *E. coli* FabG [16, 19, 20] are present in both *R. solanacearum* proteins (Fig. 1b). Moreover, two *E. coli* FabG residues, Arg-129 and Arg-172, reported to play critical roles in facilitating the binding of the ACP moiety of the substrate [21], are conserved in RsFabG1 and RsFabG2 (Fig. 1b). Based on these criteria, it seemed reasonable to hypothesize that *RsfabG1* and *RsfabG2* both encode functional 3-ketoacyl-ACP reductases and that both play important roles in fatty acid synthesis and growth in *R. solanacearum*.

### Complementation of an *E. coli fabG* temperature-sensitive mutant with the *RsfabG* genes

**Fig. 2:**
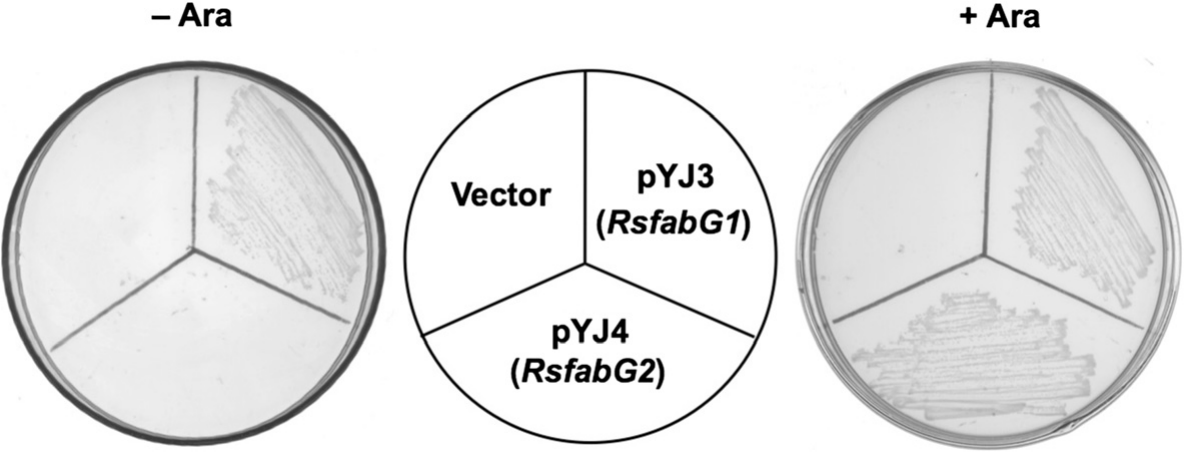
Growth of transformants of *E. coli fabG*(Ts) mutant strain CL104 with plasmids carrying the *R. solanacearum fabG*s. *E. coli* strain CL104, carrying the pBAD24M-derived plasmids pYJ3 (*RsfabG1*) and pYJ4 (*RsfabG2*), was grown at 42 °C. ARA, abbreviation of arabinose

*E. coli* strain CL104 is a *fabG* temperature-sensitive mutant [22] that lacks 3-ketoacyl-ACP reductase activity at 42 °C and is unable to grow at this non-permissive temperature. To test the functionality of RsFabG1 and RsFabG2 *in vivo*, each of the genes was inserted into the arabinose-inducible vector pBAD24M [11] to give the expression constructs pYJ3 (*RsfabG1*) and pYJ4 (*RsfabG2*). The plasmids were transferred into strain CL104 at the permissive temperature and the resulting transformants were tested for their growth at the non-permissive temperature. Strain CL104 carrying plasmid pYJ3 grew at 42 °C even in the absence of arabinose (Fig. 2). In contrast, the pYJ4-containing strain grew at 42 °C in the presence of arabinose but failed to grow in the absence of arabinose. Therefore, both of the RsFabG candidates could complement the *E. coli fabG*(ts) strain, indicating that each protein catalyzes 3-ketoacyl-ACP reduction. However, it appears likely that RsFabG2 is less active than RsFabG1 because high-level induction by arabinose was required for robust growth.

### Expression and purification of the *R. solanacearum* FabGs

**Fig. 3:**
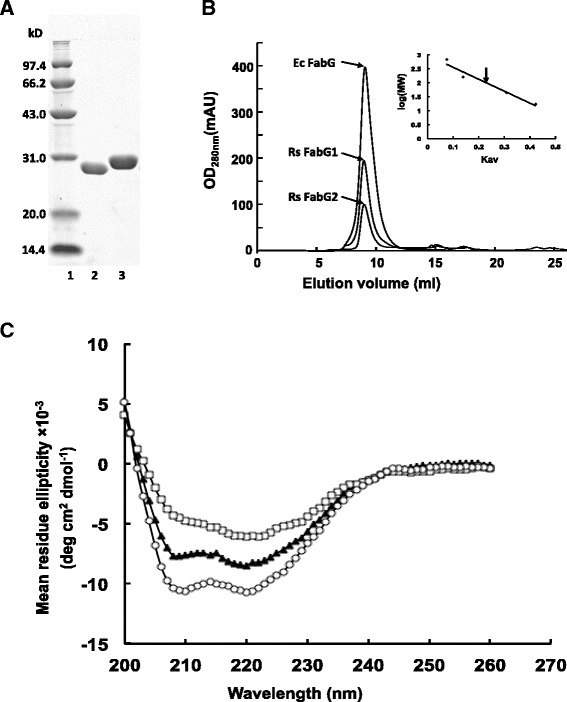
Purification of *R. solanacearum* FabGs from *E. coli* strain BL21 (DE3) and circular dichroism spectra of *R. solanacearum* FabG1 and FabG2. Panel **a**, Purification of *R. solanacearum* GMI1000 FabG1 and FabG2 by native nickel-chelate chromatography. Lane 1, molecular mass markers; lane 2, *R. solanacearum* FabG1 protein; lane 3, *R. solanacearum* FabG2 protein. Panel **b**, Size exclusion chromatography of the hexahistidine-tagged wild type RsFabG1 and FabG2 protein. The elution peaks of the molecular weight standards are given at the top of the panel. Panel **c**, CD spectra of *R. solanacearum* FabGs. CD spectra were measured at 25 °C. Open circles, *E. coli* FabG; filled triangle, *R. solanacearum* FabG1; open squares, *R. solanacearum* FabG2

To perform a direct *in vitro* assay of FabG activity, recombinant N-terminal hexahistidine-tagged RsFabGs were produced. These proteins were purified by nickel chelate chromatography to obtain preparations that gave single bands on SDS-gel electrophoresis (Fig. 3a). The purified RsFabG1 and RsFabG2 proteins have monomeric molecular weights of 29 kDa and 30 kDa, respectively. Given that *E. coli* FabG is a homo-tetramer [20], we estimated the solution structures of RsFabG1 and RsFabG2 by gel filtration chromatography (Fig. 3b). The RsFabG1 and RsFabG2 elution profiles showed that both FabGs, like *E. coli* FabG, exist as homo-tetramers in solution.

To elucidate the secondary structure and folding properties of the FabG protein, circular dichroism (CD) spectroscopy analyses were used (Fig. 3c). The CD spectrum of *R. solanacearum* FabG1, like that of *E. coli* FabG, had the characteristic helix signature with minima at 208 and 222 nm, whereas FabG2 showed only a single helix signature minimum at 222 nm. Moreover, the helical content of RsFabG2 (22.5 %) was considerably lower than that of EcFabG (33.2 %), whereas the helical content of the RsFabG1 (30.9 %) was much closer to that of EcFabG. These data indicated that, although both RsFabG1 and RsFabG2 are homo-tetramers in solution, they seem likely to have different folding patterns.

### *In vitro* enzymatic activities of RsFabG1 and RsFabG2

**Fig. 4:**
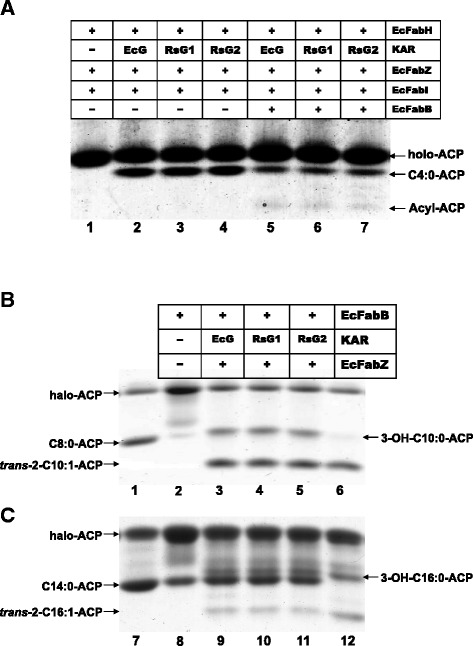
Function of *R. solanacearum* FabG1 and FabG2 in fatty acid synthesis reactions. Panel **a**, Function of *R. solanacearum* FabG1 and FabG2 in the initial cycle of fatty acid synthesis. The initial cycle of fatty acid synthesis was reconstructed *in vitro* using a combination of *E. coli* FabH (EcFabH), KAR (*E. coli* FabG (EcG) (lane 2), or *R. solanacearum* FabG1 (RsG1) (lane 3) and FabG2 (RsG2) (lane 4), *E. coli* FabZ (EcFabZ) and FabI (EcFabI) enzymes, ACP, NADH, and NADPH as cofactors and malonyl-ACP plus acetyl-CoA as substrates to produce butyryl-ACP. To complete the fatty acid synthesis reaction (lanes 5 to 7), *E. coli* FabB (EcFabB) was added to the reactions (the faint bands are due to the instability of short-chain 3-ketoacyl-ACPs in the electrophoresis gels). Panel b and c, Function of *R. solanacearum* FabG1 and FabG2 in the fatty acid elongation cycle. The elongation reaction mixture contained *E. coli* FabB (EcFabB), KAR *E. coli* FabG (EcG) (panel **b**, lane 3 or panel **c**, lane 9) or *R. solanacearum* FabG1 (RsG1) (panel **b**, lane 4 or panel **c**, lane 10), FabG2 (RsG2) (panel **b**, lane 5 or panel **c**, lane 11) and *E. coli* FabZ (EcFabZ), malonyl-ACP plus octanoyl-ACP (panel **b**) or tetradecanoyl-ACP (panel **c**) as substrates, and NADH and NADPH as cofactors. RsG1 and RsG2 denote *R. solanacearum* FabG1 and FabG2, respectively. Lane 1 is the product of octanoyl-ACP and lane 7 is the product of tetradecanoyl-ACP. Lane 6 is the product of 3-hydroxydecanoyl-ACP treated with *E. coli* FabZ and lane 12 is the product of 3-hydroxyhexadecanoyl-ACP treated with *E. coli* FabZ

The function of the two RsFabGs in fatty acid synthesis was assayed *in vitro*. First, the enzymatic activity of RsFabGs in the initial steps of fatty acid synthesis was tested as described in the Methods. In the absence of a 3-ketoacyl-ACP reductase, only holo-ACP was seen (Fig. 4a, lane 1), probably due to hydrolysis of the labile 3-ketobutyryl-ACP during electrophoresis [23]. Addition of RsFabG1, RsFabG2 or EcFabG to the reaction mixture resulted in production of butyryl-ACP (Fig. 4a, lanes 2–4). Upon addition of the long-chain *E. coli* 3-ketoacyl-ACP synthase, EcFabB, to the reactions, all reactions produced long-chain acyl-ACP species (Fig. 4a, lanes 5–7). These data clearly showed that, like *E. coli* FabG, both RsFabG1 and RsFabG2 could complete the initial cycle of fatty acid synthesis to produce butyryl-ACP.

Next, the enzymatic activity of the two RsFabGs in the reduction of long-chain 3-ketoacyl-ACP substrates was also examined. Incubation of EcFabB with malonyl-ACP and octanoyl-ACP or tetradecanoyl-ACP resulted in the formation of 3-ketodecanoyl-ACP (Fig. 4b, lane 2) or 3-ketohexadecanoyl-ACP (Fig. 4c, lane 8), respectively (the longer-chain species are more stable during electrophoresis). Upon the addition of NADPH, a 3-ketoacyl-ACP reductase (EcFabG, RsFabG1 or RsFabG2) and EcFabZ to the reaction mixture all incubations produced 3-hydroxyacyl-and enoyl-ACP species (Fig. 4b, lanes 3–5, and c lanes 9–11). Note that EcFabG converts 3-ketoacyl-ACPs to 3-hydroxacyl-ACPs, whereas *E. coli* FabZ dehydrates 3-hydroxacyl-ACPs to produce enoyl-ACPs. Therefore, these data indicate that both RsFabGs are active with long-chain 3-ketoacyl-ACP substrates, consistent with their ability to support the growth of *E. coli*.

Acetoacetyl-CoA is the substrate most often used to test 3-ketoacyl-ACP reductase activity (the cognate acyl-CoA substrate often functions in place of the acyl-ACP substrates although generally with a much higher Km value) [16]. Thus, the kinetic properties of RsFabGs (Table 1) were examined using the model substrate acetoacetyl-CoA as substrate. RsFabG1 reduced acetoacetyl-CoA, although with a lower enzyme activity than *E. coli* FabG, whereas RsFabG2 failed to reduce the model substrate (Table 1). The maximal velocities of acetoacetyl-CoA reduction of RsFabG1 or EcFabG were strikingly different, as were the Km values of RsFabG1 and EcFabG. The maximal velocity of RsFabG1 for the NADPH substrate was lower than that of EcFabG, and the Km of RsFabG1was higher than that of EcFabG (Table 1).

**Table 1:** Kinetic parameters of *R. solanacearum* FabG proteins

	V_max_ (nmol/min × μg)		K_m_ (μmol/L)		K_cat_ (sec^−1^)	
	AAC^a^	NADPH	AAC ^*a*^	NADPH	AAC	NADPH
EcFabG	97.02 ± 8.5	3.54 ± 0.5	4372.6 ± 105.1	5.41 ± 1.3	45.28 ± 4.3	1.65 ± 0.43
RsFabG1	4.70 ± 1.3	1.50 ± 0.5	313.28 ± 14.5	25.75 ± 3.8	2.19 ± 078	0.71 ± 0.24
RsFabG2	ND ^*b*^	ND	ND	ND	ND	ND

^*a*^ AAC denotes acetoacetyl-CoA

^*b*^ ND, could not be detected

### Essentiality of the *R. solanacearum RsfabG1* gene

To examine whether the two 3-ketoacyl-ACP reductases are essential for *R. solanacearum* cell growth and cellular fatty acid synthesis we attempted disruption of both genes. Plasmids containing pK18mobsacB-borne Gm resistance cassette insertions in *RsfabG1* (pYJ27) or *RsfabG2* (pYJ30) were constructed. Plasmids (pYJ27 or pYJ30) carrying the gene disruptions were introduced into the genome of wild type *R. solanacearum* strain GMI1000 via conjugal transfer from *E. coli* S17-1. Mutant colonies were subsequently screened on a medium containing sucrose and gentamicin. The success of the mutants was assayed by colony PCR analysis using two primer pairs listed in Additional file 1: Table S2. During our attempts to generate an *RsfabG1*-disrupted *R. solanacearum* strain, only single crossover integrants were obtained (Additional file 2: Figure S1, A). This result suggested that *RsfabG1* is an essential *R. solanacearum* gene.

To test this hypothesis we constructed plasmid pYJ33, which carries *RsfabG1* under the control of the *E. coli lac* promoter, and transformed this plasmid into an *RsfabG1* single crossover integrant stain (Additional file 2: Figure S1, A). The transformants were then plated on medium containing gentamicin, sucrose and IPTG to select for loss of *sacB* function. Colony PCR assays using the primer pair RsFabG1 upside and RsFabG1 downside showed that one of the surviving colonies, named strain RS-G5 (*RsfabG1*::Gm/pYJ33), in which *RsfabG1* was replaced by the gentamicin resistance gene, contained the defined 2.0 kb Gm resistance-containing fragment. As expected, a 2.3 kb *RsfabG1*-containing fragment was amplified from the wild type strain using the same primer pair (Additional file 3: Figure S2, A). Using primer pair RsFabG1ck1 and RsFabG1ck2, colony PCR amplified a 0.7 kb RsfabG1-containing fragment from both the wild type and RS-G5 (*RsfabG1::Gm*/pYJ33) strains because Rs-G5 (*RsfabG1::Gm*/pYJ33) contains the plasmid-borne pYJ33 copy of *RsfabG1* (Additional file 3: Figure S2, B). Insertion of the Gm cassette plus deletion of *RsfabG1* sequences was also confirmed by sequencing of the PCR fragment of the disruption allele.

Moreover, we constructed pYJ32, a pK18mobsacB-borne plasmid containing *E. coli fabG* plus a downstream Gm resistance cassette. Plasmid pYJ32 was transformed into the wild type strain GMI1000 and the transformants were processed through the counter-selection protocol described above (Additional file 2: Figure S1, B). This gave strain RS-G3 (*RsfabG1::EcfabG*) in which *RsfabG1* had been replaced by *E. coli fabG* as shown by colony PCR analysis (Additional file 3: Figure S2, A and B) with same primer pairs as used above (Additional file 1: Table S2).

Next, the strains lacking a functional chromosomal copy of *RsfabG1*, including RS-G5 (*fabG1*::Gm/pYJ33) and RS-G3 (*fabG1*::*EcfabG*), were tested for growth in M63 medium at 30 °C. Under IPTG induction, strain RS-G5 (*fabG1*::Gm/pYJ33) grew in M63 medium, although it had a generation time of 256 ± 10 min which is appreciably longer than that of the wild type *R. solanacearum* strain GMI1000 (150 ± 7 min), but RS-G5 (*fabG1*::Gm/pYJ33) failed to grow without IPTG induction. However, the *E. coli* FabG substitution strain RS-G3 (*RsfabG1*::*EcfabG*), which had a generation time of 157 ± 4 min, grew as well as the wild type strain. Collectively, these data demonstrated that *RsfabG1* is an essential gene in *R. solanacearum* and that its function can be replaced by the *E. coli fabG* gene.

As assayed by gas chromatography–mass spectrometry (GC-MS), though both mutants contained the same fatty acid species as the wild type strain on M63 medium plates, the two mutant strains all produced more unsaturated fatty acids (UFAs) and fewer 3-hydroxy fatty acids (3-HFAs) than the wild type strain GMI1000 (Table 2). The ratios of UFAs to SFAs in strains RS-G3 (*fabG1*::*EcfabG*), and RS-G5 (*fabG1*::Gm/pYJ33) were 1.09 and 1.29, respectively, much higher than that of the wild type strain (0.57). In addition, the ratios of 3-hydroxy fatty acids to non-hydroxyl fatty acids in these strains were 0.35, and 0.44, respectively, lower than that of the wild type strain (0.66).

**Table 2:** Fatty acid composition of total lipid extracts from *R. solanacearum fabG* mutant strains grown on M63 medium plates ^*a*^

Fatty acid	RS-G2 (*fabG2*::Gm)	RS-G3 (*fabG1*::*EcfabG*)	RS-G5 (*fabG1*::Gm/pYJ33)	GMI1000
n-C14:0	2.32 ± 0.09	3.00 ± 0.10	2.99 ± 0.13	2.38 ± 0.40
n-C14:0 3-OH	32.53 ± 2.24	21.49 ± 0.28	29.68 ± 2.54	39.00 ± 2.87
n-C16:1 *cis* 9	11.54 ± 1.16	16.88 ± 0.48	20.82 ± 1.50	10.52 ± 1.11
n-C16:0	23.25 ± 1.25	28.71 ± 1.94	22.76 ± 1.75	28.47 ± 3.74
n-C16:0 2-OH	2.14 ± 0.44	1.59 ± 0.09	1.03 ± 0.12	2.06 ± 0.16
n-C17:0 cyclo	5.76 ± 0.73	5.51 ± 0.74	1.14 ± 0.24	1.89 ± 0.17
n-C18:1 *cis* 11	17.20 ± 2.43	17.37 ± 2.12	16.96 ± 0.67	8.98 ± 0.96
n-C18:0	4.21 ± 0.36	4.62 ± 0.29	4.31 ± 0.29	6.54 ± 0.89
UFA/SFA^*b*^	1.16	1.09	1.29	0.57
HFA/FA	0.57	0.35	0.44	0.70

^*a*^Cells were grown on M63 plates at 30 °C for two days. The total lipids were extracted and transesterified to obtain fatty acid methyl esters, and products were identified by GC-MS. The values are the means ± standard deviations of three independent experiments and the percentages of total fatty acids

^*b*^UFA denotes unsaturated fatty acid, including C16:1 *cis* 9, C17:0 cyclo and C18:1 *cis* 11. SFA denotes saturated fatty acid, including C14:0, C16:0 and C18:0. HFA denotes hydroxyl-fatty acid, including C14:0 3-OH and C16:0 2-OH

### Effects of *RsfabG2* mutation on growth of *R. solanacearum*

Using a similar approach, we readily obtained a *RsfabG2* Gm insertion mutant (named strain RS-G2 (*RsfabG2::Gm*)) (Additional file 2: Figure S1, C). The growth of Rs-G2 (*fabG2*::Gm) in M63 medium was tested. In M63 medium at 30 °C, Rs-G2 (*RsfabG2::Gm*) had a generation time of 170 ± 5 min, somewhat longer than that of the wild type strain (150 ± 7 min), indicating that *RsfabG2* was not essential for *R. solanacearum* growth.

The GC-MS assays showed that strain Rs-G2 (*RsfabG2::Gm*) also contained significantly more UFAs and fewer 3-HFAs than the wild type strain (the UFA/SFA ratio for Rs-G2 (*RsfabG2::Gm*) was 1.16; the HFA/FA ratio was 0.57) (Table 2). However, in the RS-G2 strain the major increase in UFAs was in C_18:1_ species, whereas both C_16:1_ and C_18:1_ species of UFA were increased in *RsfabG1* mutant strains (RS-G3 and RS-G5). This suggested that, like RsFabG1, RsFabG2 plays a role in determining the cellular fatty acid profile of *R. solanacearum* and that some changes in 3-ketoacyl-ACP reductases affect the fatty acid composition of *R. solanacearum*.

**Fig. 5:**
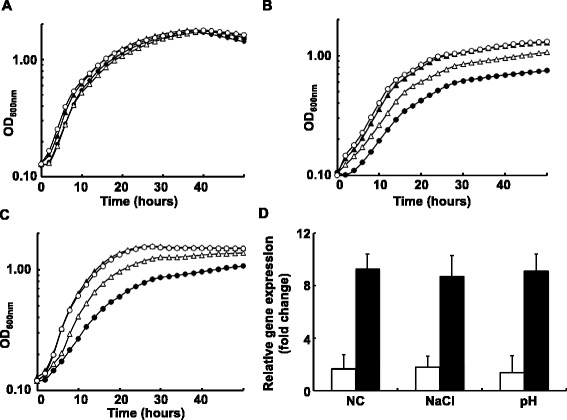
Growth of the *R. solanacearum fabG2* mutant strain in BG medium under high salt or low pH conditions. Panel **a**, The growth of mutant strain RS-G2 in BG medium under natural conditions (30°C, pH 7.0 and no addition of NaCl). Panel **b**, The growth of RS-G2 in BG medium containing 0.1 M NaCl at 30°C. Panel **c**, The growth of RS-G2 in BG medium with pH adjusted to 5.5 by 1 N HCl at 30°C. Filled circle denotes RS-G2 strain, empty triangle denotes RS-G2/pYJ34 strain, filled triangle denotes RS-G2/pYJ35 strain and empty circle denotes GMI1000 strain. Panel **d**, Expression of* RsfabG1* and *RsfabG2* under hight salt or low pH conditions. Open bar denotes *RsfabG1* and filled black bar denotes *RsfabG2*.

In order to investigate possible physiological functions of RsFabG2, we also tested the growth of strain RS-G2 (*RsfabG2::Gm*) in BG medium under various environmental challenges (high temperature, high salt concentration or low pH). Although the growth of strain RS-G2 (*RsfabG2::Gm*) was similar to that of the wild type strain at different temperatures (data not shown), high salt concentration (0.1 M NaCl) or low pH (pH 5.5) differentially affected the growth of strain RS-G2 (*RsfabG2::Gm*) (Fig. 5b and c). Note that under natural conditions (pH 7.0 without addition of NaCl) all strains grew well (Fig. 5a). Under these conditions, RS-G2 (*RsfabG2::Gm*) grew more slowly than the wild strain GMI1000 (Fig. 5b and c). Complementation of RS-G2 (*RsfabG2::Gm*) with plasmid pYJ34, which carries wild type *RsfabG2*, restored the growth of RS-G2 (*RsfabG2::Gm*) (Fig. 5). These results indicated that, although *RsfabG2* is not an essential gene in *R. solanacearum*, it does play a role in the adaptation of *R. solanacearum* to environmental stress. To test whether the expression of *RsfabG2* is controlled by environmental stress, the transcript level of *RsfabG1* and *RsfabG2* was examined under high salt concentration and low pH by quantitative RT-PCR (Fig. 5d). The level of expression of *RsfabG2* was 6- to 7-fold greater than that of *RsfabG1* under all conditions, and the level of expression of *RsfabG2* under high salt concentration or low pH was almost same as that under natural conditions (Fig. 5d). These data indicate that the expression of *RsfabG1* and *RsfabG2* seems to be constitutive. We also complemented RS-G2 (*RsfabG2::Gm*) with plasmid pYJ35, which carries *E. coli* wild type *EcfabG*. This strain grew as well as strain GMI1000 under these conditions (Fig. 5). Therefore, we speculate that it is the level of activity of 3-ketoacyl-ACP reductase rather than the *RsfabG2* gene or protein product that is important for the response of *R. solanacearum* to stress. This was also observed in the pathogenesis experiments described below.

### Mutation of *RsfabG2* impaired the virulence of *R. solanacearum* in tomato plants

**Fig. 6:**
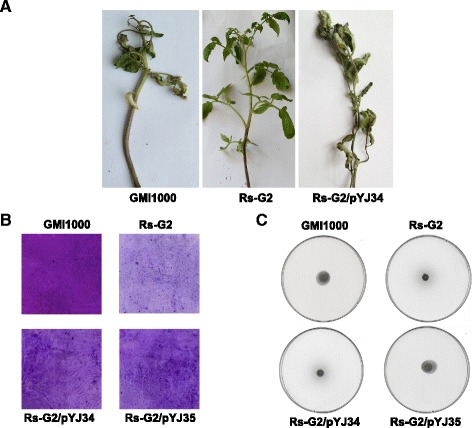
Effects of *RsfabG2* on virulence of *R. solanacearum*. Panel **a**, Pathogenicity test on tomato plants with the *R. solanacearum* GMI1000 or RS-G2 mutant strain. Panel **b**, Biofilm formation of strain RS-G2. Panel **c**, Swimming motility of the RS-G2 strain

*R. solanacearum* is a phytopathogenic bacterium that causes vascular wilt diseases in many plants [18]. To investigate the role of *RsfabG2* in the virulence of *R. solanacearum*, tomato plants were inoculated with the wild type GMI1000 and the Rs-G2 (*RsfabG2*::Gm) strain. The wild type GMI1000 strain caused symptoms of wilt on the 3rd day after inoculation and the test tomato plants showed complete wilting by 7 days after infection, whereas the RS-G2 (*RsfabG2*::Gm) strain caused no disease symptoms until 21 days after inoculation (Fig. 6a). Furthermore, both *RsfabG2* and *EcfabG* caused some tomato plant wilting and hence partially restored the virulence of the RS-G2 (*RsfabG2*::Gm) strain; restoration of *EcfabG* was more effective than that of *RsfabG2*. The mortality rates of tomato plants infected by these strains throughout the course of the infection were: GMI1000, 100 %; RS-G2 (*RsfabG2*::Gm), 0 %; RS-G2/pYJ34, 40 % and RS-G2/pYJ35, 65 %.

We also evaluated several pathogenicity-related phenotypes of strain RS-G2 (*RsfabG2*::Gm). The production of EPS I (extracellular polysaccharide I), EGL (endoglucanase), and PGL (polygalacturonase) by RS-G2 (*RsfabG2::Gm*) was first tested. The data showed that production of EPS I, EGL, and PGL was not significantly impaired in the mutated strain RS-G2 (*RsfabG2*::Gm) (data not shown). Next, the biofilm formation and swimming motility of strain RS-G2 (*RsfabG2*::Gm) were examined. After *R. solanacearum* strains had been incubated without shaking in BG broth at 28 °C for 36 h, the biofilms formed on the slides were quantified. It was found that RS-G2 (*RsfabG2*::Gm) produced less biofilm (OD_530_, 0.02 ± 0.01) than the wild type strain GMI1000 (OD_530_, 0.11 ± 0.02) (Fig. 6b). The swimming motility of RS-G2 (*RsfabG2*::Gm) was evaluated on semi-solid motility agar. After 2 days at 28 °C, the RS-G2 (*RsfabG2*::Gm) swimming haloes were about half as large as those formed by GMI1000. When complemented with *EcfabG*, the motility of the RS-G2 (*RsfabG2*::Gm) strain was restored to that of the wild type, whereas strain RS-G2 (*RsfabG2*::Gm) complemented with *RsfabG2* formed the same small halo as RS-G2 (*RsfabG2*::Gm) (Fig. 6c). In order to explain why complementation of RS-G2 (*RsfabG2*::Gm) with *RsfabG2* failed to restore swimming motility, the 3-ketoacyl-ACP reductase activities were tested in cell-free extracts of these strains. Although the 3-ketoacyl-ACP reductase activity (in μmol/min per mg extract protein) in strain RS-G2 carrying the *RsfabG2* encoded plasmid (0.375 ± 0.015) was higher than that in strain RS-G2 (0.250 ± 0.068), it was still lower than that in strain RS-G2/pYJ35 (0.485 ± 0.058) or the wild type strain (0.576 ± 0.056). These results indicate that the level of activity of 3-ketoacyl-ACP reductase is important for the lifestyle of *R. solanacearum* and that mutation of *RsfabG2* can impair biofilm formation and decrease swimming motility.

**Fig. 7:**
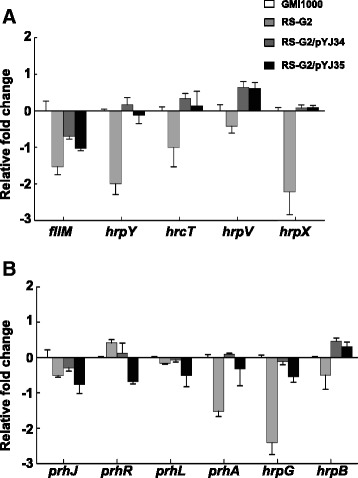
Expression of the virulence-related genes in the RS-G2 mutant. Panel **a**, Expression of the structural genes, including *fliM*, *hrpY*, *hrcT*, *hrpV* and *hrpX*, in the RS-G2 strain. Panel **b**, Expression of the regulator genes, including *prhJ*, *prhR*, *prhI*, *prhA*, *hrpG* and *hrpB*. Bacteria were cultured and RNA was extracted as described in the “Materials and Medthods”. The *rplM* gene was used as the internal control for quantitative RT-PCR. The experiment was performed at least three time using independent batches of samples with similar results. Results from a single representative sample are shown. The means ± SD (error bars) of three determinations from cDNA from a single representative sample are shown

To address the above observations further, we examined the expression of the genes related to *R. solanacearum* virulence determinants, including *epsA*, *egl*, *pehB*, *pglA*, *fliC*, *fliM*, *pilQ*, *pilT* and T3SS-related genes (*popA*, *popC*, *hrcT*, *hrpK*, *hrpY*, *hrpX* and *hrpV*), in the RS-G2(*RsfabG2::Gm*) mutant by quantitative RT-PCR. The expression of *fliM*, *hrpY*, and *hrpX* was obviously decreased in the RS-G2 (*RsfabG2::Gm*) mutant compared with the wild type strain GMI1000 (p < 0.001) (Fig. 7a), whereas the level of expression of the remaining virulence-related genes in RS-G2 (*RsfabG2*::Gm) was not significantly different from that in the wild type strain (p > 0.05) (data not shown). We also determined the levels of expression of six genes (*hrpB*, *hrpG*, *prhJ*, *prhR*, *prhI* and *prhA*) in a multigene regulatory cascade responsible for transcription of the entire *hrp* regulon. When compared with the wild type strain GMI1000, the results showed that the expression level of *prhA* and *hrpG* was significantly reduced in the RS-G2 (*RsfabG2::Gm*) mutant (p < 0.001), and the expression of *prhJ* and *hrpB* was slightly lower in the RS-G2 (*RsfabG2*::Gm) mutant than in GMI1000, while the level of *prhR* and *prhI* in RS-G2 (*RsfabG2*::Gm) was almost the same as in the wild type strain (p > 0.05) (Fig. 7b). Meanwhile, both the *RsfabG2* and the *EcfabG* genes could restore the expression of these genes in RS-G2 (*RsfabG2*::Gm) to the level of the wild type strain. These data indicated that impairment of *R. solanacearum* virulence by deletion of *RsfabG2* seems to be due to reduction of the expression of various genes that are related to virulence determinants.

## Discussion

In bacterial fatty acid synthesis, 3-ketoacyl-ACP reductase (KAR) catalyzes the key reduction of 3-ketoacyl-ACPs to 3-hydroxy acyl-ACPs [3]. In *E. coli*, *fabG* is the only gene that encodes 3-ketoacyl-ACP reductase activity [21, 22]. However, in other species of bacteria, multiple *fabG* paralogues have been annotated. For example, the genome of *Mycobacterium tuberculosis* has five annotated *fabG* genes [24]. Although *M. tuberculosis fabG1* and *fabG4* both complement a yeast *Δoar1* mutant strain (which lacks the mitochondrial 3-KAR activity), there is no other evidence for a bacterium that has more than one functional KAR. It should be noted that genomes generally encode many SDR family members and thus, in the absence of a genomic context consistent with a role in fatty acid synthesis, a true KAR can be very difficult to distinguish from other SDR family members catalyzing unrelated reactions, such as reduction of the keto group of an intermediate in sugar utilization. Moreover, even when a plausible genomic context is present, misannotation can occur, as was the case in *Lactococcus lactis* where a putative KAR lacked activity both *in vivo* and *in vitro* [17].

Growth of *R. solanacearum* requires RsFabG1, but not RsFabG2. The *RsfabG1* gene is found within a highly conserved chromosomal gene cluster that apparently encodes several enzymes required for fatty acid synthesis, whereas the *RsfabG2* gene is located on the *R. solanacearum* megaplasmid within a gene cluster containing *fabB*, *fabF2* and *fabF3* [18]. Our prior studies have shown that the protein products encoded by the megaplasmid genes *fabB*, *fabF2* and *fabF3* do not play roles in fatty acid synthesis [25], but we found that *RsfabG2* complemented growth of the *E. coli fabG*(ts) strain CL104 and catalyzed all of the keto reduction steps in the elongation cycle of FAS II *in vitro*. Moreover, the strain in which *RsfabG2* had been disrupted remained viable, although the mutant strain contained significantly more UFAs than the wild type strain, and was sensitive to a high salt concentration and low pH. This result indicated that the *RsfabG2* gene plays a role in growth rate and in adaptation to environmental stress. Furthermore, deletion of *RsfabG2* reduced the ability of *R. solanacearum* to form a biofilm and impaired *R. solanacearum* swimming motility and the pathogenesis of disease in tomato plants. It should be noted that the *R. solanacearum* megaplasmid could reasonably be considered a second chromosome because a number of genes of primary metabolic pathways, including amino acid and cofactor biosynthesis, are encoded on the megaplasmid [18]. The megaplasmid also carries all of the *hrp* genes required to cause plant disease and encodes the constituents of the flagellum and most of the genes governing exopolysaccharide synthesis. This bipartite genome structure is characteristic of most *R. solanacearum* strains [26], and derivatives of strain GMI1000 lacking the megaplasmid have not been obtained [18]. Finally bioinformatic analyses indicate that the two replicons have shared a similar evolutionary history, suggesting that the megaplasmid was not recently acquired from another organism by lateral gene transfer but is rather a part of an ancestral *R. solanacearum* chromosome [27]. Therefore, the presence of a megaplasmid gene encoding a functional fatty acid synthetic enzyme and playing a role in virulence in tomato plants does not seem atypical, although this raises the question of the functions of the gene cluster (*fabF3* RSp0360 *fabG2 fabF2 fabB*).

Although the protein products encoded by *fabB*, *fabF2* and *fabF3* do not play roles in fatty acid synthesis, FabB, FabF2 and FabF3 indeed display higher identities with *E. coli* FabF, one of the long-chain 3-ketoacyl-ACP synthases [25]. Together with our observation about RsFabG2 in this report, we speculate that the gene products of this cluster (*fabF3* RSp0360 *fabG2fabF2 fabB*) may constitute a novel pathway to synthesize an unidentified signal that is related to fatty acids, which may induce *R. solanacearum* pathogenesis in plants. A similar scenario has been investigated in *Sinorhizobium meliloti*, in which the *nodFEG* gene cluster was essential for the production of unusual α, β-unsaturated fatty acid moieties of the Nod factors that trigger nodule formation on the roots of alfalfa. The gene products of *nodF*, *nodE* and *nodG* have recognizable homologies to acyl carrier protein (ACP), 3-ketoacyl-ACP synthase and 3-ketoacyl-ACP reductase, respectively. Therefore, if the enzymes encoded by the gene cluster (*fabF3* RSp0360 *fabG2fabF2 fabB*) are required to synthesize a specific signal that induces *R. solanacearum* pathogenesis in plants, it will be easy to understand why deletion of *RsfabG2* from the megaplasmid caused the expression of virulence-related genes to decrease and reduced *R. solanacearum* pathogenesis in tomato plants.

Meanwhile, although both *RsfabG2* and *EcfabG* partially complemented the *R. solanacearum fabG2* mutation, *EcfabG* restored it more effectively than *RsfabG2* itself. This suggested that the overall cellular level of 3-ketoacyl-ACP reductase activity is important for the *R. solanacearum* lifestyle. Rhizobia present a similar scenario. The *nodG* gene is located in the *nodFEG* operon on the symbiotic plasmid of many rhizobia. It has been demonstrated that, although the NodG of *R. leguminosarum* has 3-ketoacyl-ACP reductase activity *in vitro* [28], the *fabG* gene cannot be deleted from the chromosome of *S. meliloti*. However, our experiments showed that, upon overexpression of *nodG* from plasmid pSRK-Gm, the *fabG* gene could be disrupted and the mutant strain was not defective in growth and fatty acid synthesis (data not published). This means that a high level of activity of 3-ketoacyl-ACP reductase is also essential to the growth of *S. meliloti*.

We also tried to disrupt *RsfabG1* in strains where *RsfabG2* was overexpressed from a plasmid, but our attempts were unsuccessful. Although the reason for this failure is unknown, it suggests that *RsFabG2* and *RsFabG1* are designed for distinct physiological roles. Although the catalytically active SDR triad, the N-terminal cofactor binding sequence [16, 19, 20], and two Arg residues important in binding the ACP moiety of the physiological substrate [21] are conserved in the two proteins (Fig. 1c), RsFabG2 is only 41 % identical to RsFabG1. Moreover, RsFabG1 reduced acetoacetyl-CoA whereas RsFabG2 could not. We investigated whether poor expression of *RsfabG2* explains its inability to bypass loss of *RsfabG1*. This does not appear to be the case, because *RsfabG2* transcript levels were 6- to 7-fold greater than those of *RsfabG1* in both log phase and stationary phase cultures (data not shown).

## Conclusion

In the bacterial fatty acid synthesis pathways studied to date only a single enzyme, FabG, has been found to catalyze the reduction of 3-ketoacyl-ACPs to 3-hydroxy acyl-ACPs. However, in this report we supply the first evidence that *R. solanacearum* encodes two functional FabG homologues. *RsfabG1* is essential for growth of *R. solanacearum*, whereas *RsfabG2* plays roles in determining the cellular fatty acid composition, adaptation to two environmental stresses and in the pathogenesis of disease in tomato plants. Therefore, like other enzymes in the bacterial fatty acid biosynthetic pathway, one bacterium may simultaneously possess two or more 3-oxoacyl-ACP reductase isozymes that have functions in fatty acid synthesis.

## Methods

### Bacterial strains, plasmids and growth media

The *E. coli* K-12 strains, *R. solanacearum* strains, and plasmids used in this study are listed in Additional file 4: Table S1. The *R. solanacearum* strains were routinely grown at 30 °C in BG broth or on BG agar (BG plus 1.6 % agar) [25]. Luria-Bertani (LB) medium was used as the rich medium for *E. coli*. The phenotypes of *E. coli fab* strains were assessed on rich broth (RB) medium [29]. M63 medium [30] supplemented with 0.1 % Casamino Acids was used to screen *R. solanacearum* mutants and, if needed, 5 or 10 % sucrose was added. Antibiotics were used at the following concentrations (in μg/ml): sodium ampicillin, 100; kanamycin sulfate, 30; chloramphenicol, 30; and gentamicin sulfate, 10 (for *E. coli*) or 30 (for *R. solanacearum*). L-Arabinose was used at a final concentration of 0.01 %. Isopropyl-β-D-thiogalactoside (IPTG) was used at a final concentration of 1 mM, and 5-bromo-4-chloro-3-indolyl-β-D-galactoside (X-Gal) was used at a final concentration of 20 μg/ml.

### Recombinant DNA techniques and construction of plasmids

To obtain the *R. solanacearum fabG* genes, genomic DNA was extracted from strain GMI1000 using the Takara DNA extraction kit. The genomic DNA was then used for PCR amplification with *Pfu* DNA polymerase and the primers listed in Additional file 1: Table S2, and the products were inserted into T-vector plasmid pMD19 to give plasmids pYJ1 (*RsfabG1*) and pYJ2 (*RsfabG2*). The *fabG* sequences were confirmed by sequencing done by Shanhai Sangon, Inc. To produce plasmids pYJ3 (*RsfabG1*), pYJ4 (*RsfabG2*), pYJ5 (*RsfabG1*) and pYJ6 (*RsfabG2*), the T-vector pMD19 *fab* gene plasmids were digested with NdeI and HindIII. The fragments were gel purified and ligated into either pBAD24M [11] or pET-28b digested with the same enzymes. The NdeI–HindIII fragments from pYJ1 (*RsfabG1*) and pYJ2 (*RsfabG2*) were ligated into pSRK-Tc or pSRK-Km digested with the same enzymes to yield pYJ33 (*RsfabG1*) and pYJ34 (*RsfabG2*), respectively. Meanwhile, we also constructed pYJ35, in which *E. coli fabG* was inserted into NdeI and HindII sites of pSRK-Km.

### Disruption and essentiality testing of the *RsfabG* genes

To disrupt *R. solanacearum fabG1*, a suicide plasmid was constructed as follows. The 500 bp DNA fragments located upstream and downstream of *RsfabG1* (called Up fabG1 and Down fabG1, respectively) were amplified with *Pfu* DNA polymerase using *R. solanacearum* genome DNA as the template and either RsFabG1 Knt up EcoRI and RsFabG1 Knt up 2 (for Up fabG1) or RsFabG1 Knt down 1 and RsFabG1 Knt down HindIII (for Down fabG1) as primers (Additional file 1: Table S2). The products of these PCR reactions were purified, and overlap PCR was carried out using RsFabG1 Knt up EcoRI and RsFabG1 Knt down HindIII as the primers. The resulting 1000 bp DNA fragment was digested with EcoRI and HindIII and inserted between the same sites of pHSG399 [31] to yield pYJ25. A 500 bp gentamicin resistance cassette was also amplified from plasmid p34s-Gm [32] with Gm up BspHI plus Gm down XbaI (Additional file 1: Table S2) as the primers. The PCR product was digested with BspHI and XbaI and cloned between the same sites of pYJ25 to give pYJ26. The 1500 bp *fabG1*:: Gm fragment of pYJ26 was digested with EcoRI and HindIII and ligated into the same sites of pK18mobsacB [33] to yield pYJ27. In the same manner we constructed pYJ30, which carried a 1500 bp *fabG2*:: Gm fragment. To replace *R. solanacearum fabG1* with *E. coli* CL104 *fabG* (ts) [22], a suicide plasmid was constructed as follows. The 750 bp *EcfabG* gene was amplified with *Pfu* DNA polymerase using *E. coli* CL104 genomic DNA as the template and EcFabG (ts) up and EcFabG (ts) down as primers. A 750 bp gentamicin resistance cassette was also amplified from plasmid p34s-Gm with Gmdp up plus Gmdp down XbaI (Additional file 1: Table S2) as the primers. The products of these PCRs were purified, and overlapping PCR was carried out using EcFabG (ts) up and Gmdp down XbaI as the primers. The 1500 bp DNA fragment containing *EcfabG*-Gm was digested with BspHI and XbaI and inserted into the same sites of pYJ25 to yield pYJ31. The 2500 bp PCR fragment containing the *RsfabG1*::[Φ(*EcfabG*-Gm] digested with EcoRI and HindIII from pYJ31 was inserted into pK18mobsacB to yield pYJ32.

A derivative of *E. coli* strain S17-1 carrying plasmids pYJ27 (*RsfabG1*), pYJ30 (*RsfabG2*) or pYJ32 (*EcfabG*) was mated with *R. solanacearum* GMI1000 on BG plates for 24 h at 30 °C. The cells were suspended in BG medium and appropriate dilutions were spread on BG plates containing chloramphenicol (to select against the donor strain) plus gentamicin and kanamycin to select for integration of the non-replicating plasmid into the genome of the recipient. Several colonies were inoculated into BG medium, and the cultures were incubated at 30 °C for 24 h, after appropriate dilutions were spread on BG plates containing 10 % sucrose. The resulting colonies were inoculated onto BG plates containing kanamycin or gentamicin using sterile toothpicks. Colonies resistant to gentamicin and sensitive to kanamycin were screened by colony PCR utilizing the primers listed in Additional file 1: Table S2. RS-G2, the *RsfabG2* disruption mutant, and RS-G3, the *R. solanacearum* strain in which *RsfabG1* was replaced by *E. coli fabG*(ts), were obtained. We failed to obtain a *RsfabG1* deletion strain; only *RsfabG1* merodiploids were obtained.

To disrupt the *RsfabG1* gene from the *R. solanacearum* GMI1000 chromosome, plasmid pYJ33 was introduced into the *RsfabG1* merodiploid. After selection on BG plates containing 10 % sucrose, a mutant strain RS-G5 was obtained.

### Expression and purification of plasmid-encoded proteins

The pET28b-derived plasmids carrying *RsfabG* alleles were introduced into *E. coli* strain BL21 (DE3), and the respective proteins, RsFabG1and RsFabG2, were expressed at high levels and purified as described previously [11, 13]. The enzymes were homogeneous as judged by SDS-PAGE. The *E. coli* FabD, FabH, FabG, FabZ, and FabI, *Vibrio harveyi* AasS and *E. col*i holo-ACP proteins were purified as described previously [11, 13].

The solution structures of RsFabG1 and RsFabG2 were analyzed by size exclusion chromatography on a Superdex 75 10/300 GL column (GE Healthcare) using an Äkta fast protein liquid chromatography (FPLC) system (Pharmacia) at 0.45 ml/min in phosphate running buffer (135 mM NaCl, 2.7 mM KCl, 1.5 mM Na_2_HPO_4_ and 8 mM K_2_HPO_4_, 10 % glycerol, pH 7.4) and the standards used previously [13, 34].

### Circular dichroism measurements

The circular dichroism (CD) spectra of FabGs were obtained on the Chirascan (Applied Photophysics Limited, Leatherhead, Surrey, UK) at 25 °C using a 1.0 nm bandwidth, 1 mm cell, 1.0 nm step, 0.5 dwell time and 1.0 min time internal. The CD spectra were measured at an enzyme concentration of 4 μM in 50 mM sodium phosphate buffer (pH 7.5). The results were expressed as molar ellipticity (θ) deg cm^2^ dmol^−1^. The values were normalized by subtracting the baseline recorded for the buffer under similar conditions.

### Assay of RsFabG1 and RsFabG2 activities *in vitro*

Malonyl-ACP was synthesized from holo-ACP and malonyl-CoA using *E. coli* FabD; octanoyl-ACP, tetradecanoyl-ACP, 3-hydroxydecanoyl-ACP and 3-hydroxyhexadecanoyl-ACP were synthesized from the acids, ATP and *E. coli* holo-ACP by *V. harveyi* acyl-ACP synthetase as described previously [35]. The abilities of RsFabG1 and RsFabG2 to function in the first cycle of fatty acid synthesis were assessed in reaction mixtures containing 0.1 M sodium phosphate (pH 7.0); 0.1 μg each of EcFabH, EcFabZ and EcFabI; 50 μM NADH; 50 μM NADPH; 1 mM-mercaptoethanol; 100 μM acetyl-CoA; 50 μM malonyl-ACP; and 50 μM holo-ACP in a final volume of 40 μl. To investigate the reduction of long-chain 3-ketoacyl-ACP, the reaction mixtures contained 0.1 M sodium phosphate (pH 7.0); 50 μM malonyl-ACP; 50 μM long chain acyl-ACP (octanoyl-ACP or tetradecanoyl-ACP); 0.1 μg each of EcFabB and EcFabZ; and 50 μM NADPH. The reactions were initiated by the addition of 0.1 μg KAR (3-ketoacyl-ACP reductase) (EcFabG, RsFabG1 or RsFabG2), followed by incubation for 1 h at 37 °C. The reaction products were resolved by conformationally sensitive gel electrophoresis on 17.5 % polyacrylamide gels containing a concentration of urea optimized for the separation [11]. The gel was stained with Coomassie Brilliant Blue R250.

### NADH oxidation assay

The 3-ketoacyl-ACP reductase activity was monitored using the decrease in absorbance at 340 nm by UV–vis spectrophotometry with an NADPH extinction coefficient of 6220 Mol^−1^. Each 500 μl reaction was performed in UV-transparent microcuvettes. The activity assays contained varying concentrations of NADPH, 0.1 μg of the purified native *R. solanacearum* FabG, varying substrate concentrations of acetoacetyl-CoA, and 0.1 M LiCl in a 0.1 M sodium phosphate buffer (pH 7). Kinetic constants were determined using GraphPad PRISM version 4 software. The Km values for NADPH were determined at an acetoacetyl-CoA concentration of 200 μM. The Km values for acetoacetyl-CoA were determined using 200 μM NADPH.

### Analysis of fatty acid compositions

The cellular lipid assay was adapted from that of Stead [36]. Briefly, cultures were grown on BG agar or M63 agar. Cells were harvested from plates with a sterile aluminum spatula into small screw-capped test tubes. Cellular lipids were saponified by addition of 1 ml NaOH in methanol solution (NaOH 45 g; methanol 150 ml; water 150 ml). The samples were placed in a boiling water bath for 30 min. The tubes were vortexed before and once during boiling. Fatty acids were methylated by addition of 2 ml 6 M HCl in methanol (325 ml 11.6 M HCl, plus 275 ml methanol). The samples were heated at 80 °C for 10 min and immediately cooled to below 20 °C. The fatty acid methyl esters were extracted three times with 1.25 ml petroleum ether. The samples were dried under a stream of nitrogen in a fume hood. The esters were analyzed by gas chromatography–mass spectrometry (GC-MS) as described previously [37]. The data are presented as percentages of the total fatty acids and represent the means ± standard error for three independent determinations.

### Pathogenicity tests

Pathogenicity tests on soil-grown plants were conducted as already described [38]. Briefly, healthy 19- to 21-day-old plants were inoculated by pouring a bacterial suspension onto the soil to a final density of approximately 6 × 10^8^ CFU/g soil, followed by incubation at 28 °C. Each strain tested was assessed for wilting in three independent 10-plant experiments.

### Motility assays

The motility of wild type and mutant strains was assayed on semisolid motility media containing 1 % (wt/vol) tryptone and 0.325 % (wt/vol) noble agar [39]. The plates were inoculated with a 2-μl drop of bacterial culture containing 1 × 10^6^ CFU/ml. Motility was visualized as a white halo of cells moving outward from the original inoculation site after 3 to 5 days of incubation at 28 °C.

### Biofilm assay

To quantify biofilm formation, we used a tube biofilm assay. Briefly, 0.2 ml of 2-day cultures of *R. solanacearum* were inoculated into 20 mL fresh BG broth in a 50-mL tube. A cover slide (microscope cover glass, 15 × 15 mm, sterilized with 70 % ethanol) was put into the tube. The culture was incubated without shaking for 36 h at 28 °C. Crystal violet staining and biofilm quantification by absorbance at 530 nm were performed as described previously [40].

### RNA extractions and quantitative real-time PCR analyses

The *R. solanacearum* wild type strain or its mutants were cultured using M63 medium containing 20 mM glutamate as previously described [41]. The total RNA of *R. solanacearum* was extracted using a TRIzol® Max™ Bacterial RNA Isolation Kit (Thermo Scientific). The RNAs were adjusted to a concentration of 200–500 ng/μl as measured with a NanoDrop 8000 (Thermo Scientific), and all samples were reverse-transcribed using a PrimeScript™ RT Reagent Kit with gDNA Eraser (Clontech). Quantitative real-time PCR analyses were carried out on an Applied Biosystems PRISM model 7500 Sequence Detection system with Maxima® SYBR Green qPCR Master Mix (Thermo Scientific). Relative quantitation was done by the comparative cycle threshold method using the endogenous internal control *rplM* for sample normalization as previously described [42]. The amplification program was as follows: 10 min at 95 °C; 40 cycles of 95 °C for 15 s, 57 °C for 1 min. The oligonucleotides used as primers are indicated in Additional file 5: Table S3. Three independent experiments were carried out for each strain and three technical replicate reactions of RT-PCR were used for each sample.

### Ethics statement

As no human or animal subjects were used for this work, consent and ethical approval were not required.

## Additional file

Additional file 1: Table S2. Sequences of the PCR primers used in this work. Additional file 2: Figure S1. Strategy for isolation R. solanacearum fabGs mutant strains. Additional file 3: Figure S2. PCR analysis of the genomic DNAs of R. solanacearum fabG1 and fabG2 mutant strains. Additional file 4: Table S1. Strains and plasmids used in this work. Additional file 5: Table S3. Sequences of the RT-PCR primers used in this work.

## Abbreviations

SDR: Short chain dehydrogenase/reductase
FAS: Fatty acid synthase
ACP: Acyl carrier protein
KAR: 3-ketoacyl-acyl carrier protein reductase
FabD: Malonyl-CoA:ACP transacylase
FabH: 3-ketoacyl-ACP synthase III
FabG: 3-ketoacyl-ACP reductase
FabZ: 3-hydroxyacyl-ACP dehydrase
FabI: Enoyl-ACP reductase
AasS: Acyl-ACP synthetase
CD: Circular dichroism
Km: Kanamycin
Gm: Gentamicin
Amp: Sodium ampicillin
Cm: Chloramphenicol
GC-MS: Gas chromatography–mass spectrometry
FA: Fatty acid
UFA: Unsaturated fatty acid
SFA: Saturated fatty acid
HFA: 3-hydroxy fatty acid
EPS I: Extracellular polysaccharide I
PGL: Polygalacturonase
EGL: Endoglucanase
IPTG: Isopropyl β-D-Thiogalactoside
X-Gal: 5-bromo-4-chloro-3-indolyl-β-D-galactoside
n-C14:0: tetradecanoic acid
n-C14:0 3-OH: 3-hydroxyl-tetradecanoic acid
n-C16:1 *cis* 9: *cis*-9-hexadencenoic acid
n-C16:0: Hexadecanoic acid
n-C16:0 2-OH: 2-hydroxyl-hexadencenoic acid
n-C17:0 cyclo: *cis*-9,10-methylene hexdecanoic acid
n-C18:1 *cis* 11: *cis*-11-octadencenoic acid
n-C18:0: Octadencenoic acid
