# Supplementary material for: Ralstonia solanacearum fatty acid composition is determined by interaction of two 3-ketoacyl-acyl carrier protein reductases encoded on separate replicons
Source: BMC Microbiol. 2015 Oct 22;15:223. doi: 10.1186/s12866-015-0554-x (PMC4618531; doi:10.1186/s12866-015-0554-x)
Supplement: Additional file 1: Table S2. — Sequences of the PCR primers used in this work. [file 12866_2015_554_MOESM1_ESM.docx]

**Table S2. Sequences of the PCR primers used in this work**

| Primer name | Primer sequence (5’ to 3’) | Digestion sites ^a^ |
| --- | --- | --- |
| RsFabG1NdeI | TTGGACCATATGACCCAAGCATTGAACAAC | NdeI |
| RsFabG1HindIII | CCGATTAAGCTTATCCCATGTACATGCCG | HindIII |
| RsFabG2NdeI | GGAAGCCATATGAGCCGCCGCGTTCTCG | NdeI |
| RsFabG2HindIII | CCCAGCAAGCTTCACACCATCCCGCCATTG | HindIII |
| Gm up BspHI | AGTAGCTCATGATGTTACGCAGCAGCAACG | BspHI |
| Gm down XbaI | CGTTGGTCTAGACGGTGGCGGTACTTG  GGTCGATATC | XbaI |
| RsFabG1 Knt up EcoRI | ATCCATGAATTCGAGAAGGCTTGTGAGATC | EcoRI |
| RsFabG1 Knt up2 | TCTAGAGTGATCTCATGAGGATTCCGTTATTTG | XbaI, BspHI |
| RsFabG1 Knt down1 | TCATGAGATCACTCTAGAGAAGCCGTCCCGGCGGCGATGTCATCGATG | BspHI, XbaI |
| RsFabG1Kntdown HindIII | CCTTCTAAGCTTCCTGCGACTCTATATCAGC | HindIII |
| RsFabG2 Knt up EcoRI | ATTCCGGAATTCGATGAACATGAACGACCGC | EcoRI |
| RsFabG2 Knt up2 | TCTAGACACTGCTCATGATGCGAAACTCCCTTC | XbaI, BspHI |
| RsFabG2 Knt down1 | TCATGAGCAGTGTCTAGATGAAGCGTGT  GGTCGTG | BspHI, XbaI |
| RsFabG2Kntdown HindIII | ATACGCAAGCTTGGCATCATCTGGATGTAAC | HindIII |
| EcFabG(Ts) up | CATCAGTCATGAATTTTGAAGGAAAAATC | BspHI |
| EcFabG(Ts) down | GCTCCATAACATCTCAGACCATGTACATCCCGC |  |
| Gmdq up | GCGGGATGTACATGGTCTGAGATGTTATGGAGC |  |
| Gmdq down XbaI | TCTCGTCTAGAACGAATTGTTAGGT | XbaI |
| RsFabG1 up | AACTGAATTCATGAGCCGCCGCGTTC |  |
| RsFabG1 down | ACTGAAGCTTCACACCATCCCGCCATTG | HindIII |
| RsFabG1 ck1 | CTCTCGTCACCGGCGCATCG |  |
| RsFabG1 ck2 | GTTGACGTGCAGCGTCGC |  |
| RsFabG1 upside | GAGCGATCGCGTTCAAGGAT |  |
| RsFabG1 downside | GAATTTGGTGACGGTGGCGA |  |
| RsFabG2 upside | TCGCGTACAGCGTATCGAACAC |  |
| RsFabG2 downside | AATGGAAGCACGGCTGATGGCA |  |

*^a^* underlined nucleotide sequences are digestion sites of restriction endonuclease.
