# Supplementary material for: Ralstonia solanacearum fatty acid composition is determined by interaction of two 3-ketoacyl-acyl carrier protein reductases encoded on separate replicons
Source: BMC Microbiol. 2015 Oct 22;15:223. doi: 10.1186/s12866-015-0554-x (PMC4618531; doi:10.1186/s12866-015-0554-x)
Supplement: Additional file 2: Figure S1. — Strategy for isolation R. solanacearum fabGs mutant strains. [file 12866_2015_554_MOESM2_ESM.docx]

**Fig. S1**

**Fig. S1. Strategy for isolation *R. solanacearum* *fabG*s mutant strains. Panels A, isolation mutant strain RS-G5 (*fabG1*::Gm/pYJ33). Panel B, isolation mutant strain RS-G3 (*fabG1*::*EcFabG*). Panels C, isolation mutant strain RS-G2 (*fabG2*::Gm).** Abbreviations: CH, chromosome; MP, megaplasmid; Up1, the upstream fragment of *fabG1*; Dn1, the downstream fragment of *fabG1*; Up2, the upstream fragment of *fabG2*; Dn2, the downstream fragment of *fabG2*.
