# Supplementary material for: Ralstonia solanacearum fatty acid composition is determined by interaction of two 3-ketoacyl-acyl carrier protein reductases encoded on separate replicons
Source: BMC Microbiol. 2015 Oct 22;15:223. doi: 10.1186/s12866-015-0554-x (PMC4618531; doi:10.1186/s12866-015-0554-x)
Supplement: Additional file 3: Figure S2. — PCR analysis of the genomic DNAs of R. solanacearum fabG1 and fabG2 mutant strains. [file 12866_2015_554_MOESM3_ESM.docx]

**Fig. S2**

**Fig. 2S. PCR analysis of the genomic DNAs of *R. solanacearum fabG1* and *fabG2* mutant strains.**

Panels A and B, PCR analysis of *fabG1* mutant strains RS-G3 and RS-G5 using primers RsFabG1 upside and RsFabG1 downside or by primers RsFabG1 ck1 and RsFabG1 ck2, respectively. Lanes 1 and 5, wild type; lanes 2 and 6, strain RS-G3; lanes 3 and 7, strain RS-G5. Panels C and D, PCR analysis of *fabG2* mutant strains RS-G2 and RS-G4 using primers RsFabG2 upside and RsFabG2 downside (A) or by primers RsFabG2 NdeI and RsFabG2 HindIII (B). Lanes 1 and 4, strain RS-G2; lanes 2 and 5, wild type.
