# Supplementary material for: Ralstonia solanacearum fatty acid composition is determined by interaction of two 3-ketoacyl-acyl carrier protein reductases encoded on separate replicons
Source: BMC Microbiol. 2015 Oct 22;15:223. doi: 10.1186/s12866-015-0554-x (PMC4618531; doi:10.1186/s12866-015-0554-x)
Supplement: Additional file 4: Table S1. — Strains and plasmids used in this work. [file 12866_2015_554_MOESM4_ESM.docx]

**Table S1. Strains and plasmids used in this work.**

| **Strains and plasmids** | **Relevant Characteristics*^a^*** | **Source** |
| --- | --- | --- |
| **Strains** |  |  |
| ***E. coli* strains** | | |
| **DH5α** | *F^-^ deoR*, *endA1hsdR17* (rk-mk+)*recA1* ∆(l*acZYA*- *argF*)U169n (ϕ80l*acZ∆M15*) | TaKaRa |
| **Top 10** | *F^-^*∆ (*lac*)X74*deoRrecA1araD139*∆(*ara-leu*)*7697endA1*(ϕ80*lacZ*del*M15*) | TaKaRa |
| **BL21 (DE3)** | *F^-^dcmompThsdS*(rB-mB-)(λDE3) | TaKaRa |
| **S17-1** | F-, *thi*, *pro*, *hsdR*, [RP4-2 Tc::Mu Km::Tn7 (Tp Sm)] | CGSC |
| **CL104** | *fabG* (Ts) *panD* Cm^r^, Tet^r^, Km^r^ | [1] |
| ***R. solanacearum* strains** | | |
| **GMI1000** | Wild type | ATCC |
| **RS-G2** | GMI1000 *fabG2*::Gm^r^ | This study |
| **RS-G3** | GMI1000 *fabG1*::Φ(Ec*fabG*-Gm resistance cassette) | This study |
| **RS-G5** | GMI1000 *fabG1*::Gm^R^/pYJ33 | This study |
| **Plasmids** | | |
| **pBAD24M** | Amp^r^; pBAD24 NcoI site replaced by NdeI site | [2] |
| **pSRK-Km** | pBBR1MCS-2-derived broad-host-range expression vector containing *lac*  promoter and *lacI^q^*, *lacZα^+^*, and Km^r^ | [3] |
| **pSRK-Tc** | pBBR1MCS-2-derived broad-host-range expression vector containing *lac*  promoter and *lacI^q^*, *lacZα^+^*, and Tc^r^ | [3] |
| **pHWG**  **pMD19** | Amp^r^, pBAD24M carrying *E. coli fabG*  Amp^r^, T-vector | This study  Takara |
| **pET28b** | Km^r^, expression vector | Novagen |
| **pHSG399** | Cm^r^ cloning vector | [4] |
| **pK18mobsacB** | Km^r^; *sacB*-based gene replacement vector | [5] |
| **p34s-Gm** | Amp^r^; Gm resistance cassette-carrying vector | [6] |
| **pYJ1** | Amp^r^, 750 bp PCR DNA fragment of *R. solanacearum fabG1* inserted into pMD19 | This study |
| **pYJ2** | Amp^r^, 750 bp PCR DNA fragment of *R. solanacearum fabG2* inserted into pMD19 | This study |
| **pYJ3** | Amp^r^, *RsfabG1* in pBAD24M; pYJ1 NdeI-HindIII fragment inserted into pBAD24M (same sites) | This study |
| **pYJ4** | Amp^r^, *RsfabG2* in pBAD24M; pYJ2 NdeI-HindIII fragment inserted into pBAD24M (same sites) | This study |
| **pYJ5** | Km^r^ , *RsfabG1* in pET-28b, pYJ1 NdeI-HindIII inserted into same sites of pET-28b. | This study |
| **pYJ6** | Km^r^ , *RsfabG2* in pET-28b, pYJ2 NdeI-HindIII inserted into same sites of pET-28b. | This study |
| **pYJ25** | Cm^r^ , a 1,000 bp DNA fragment containing Up fabG1 and Down fabG1 inserted into pHSG399 between EcoRI and HindIII sites. | This study |
| **pYJ26** | Cm^r^ , Gm^r^ , the gentamicin resistance cassette inserted into pYJ25 between BspHI and XbaI sites. | This study |
| **pYJ27** | Km^r^, Gm^r^, a 1,500 bp PCR fragment containing the *RsfabG1*::Gm resistance cassette in pK18mobsacB | This study |
| **pYJ30** | Km^r^, Gm^r^, a 1,500 bp PCR fragment containing the Rs*fabG2*::Gm resistance cassette in pK18mobsacB | This study |
| **pYJ31** | Cm^r^ , Gm^r^ , a 2,500 bp PCR fragment containing the *RsfabG1*:: [Φ(Ec*fabG*(ts)-Gm resistance cassette)] in pYJ25 | This study |
| **pYJ32** | Km^r^, Gm^r^, a 2,500 bp PCR fragment containing the *RsfabG1*:: [Φ(Ec*fabG*(ts)-Gm resistance cassette)] in pK18mobsacB | This study |
| **pYJ33** | Tc^r^, pYJ1 NdeI-HindIII fragment inserted into pSRK-Tc | This study |
| **pYJ34** | Km^r^, pYJ2 NdeI-HindIII fragment inserted into pSRK-Km | This study |
| **pYJ35** | Km^r^, *E.coli fabG* inserted between NdeI and HindIII sites of pSRK-Km | This study |

*^a^* Gm, Km, Amp and Cm denote resistance to gentamicin, kanamycin, ampicillin and chloramphenicol, respectively.

**References**

1. Lai CY, Cronan JE: **Isolation and characterization of beta-ketoacyl-acyl carrier protein reductase (fabG) mutants of Escherichia coli and Salmonella enterica serovar Typhimurium.** *J Bacteriol* 2004, 186(6): 1869-1878.
2. Zhu L, Lin J, Ma J, Cronan JE, Wang H: **Triclosan resistance of Pseudomonas aeruginosa PAO1 is due to FabV, a triclosan-resistant enoyl-acyl carrier protein reductase.** *Antimicrob Agents Chemother* 2010, 54(2): 689-698.
3. Khan SR, Gaines J, Roop RM, 2nd, Farrand SK: **Broad-host-range expression vectors with tightly regulated promoters and their use to examine the influence of TraR and TraM expression on Ti plasmid quorum sensing**. *Appl Environ Microbiol* 2008, 74(16): 5053-5062.
4. Takeshita S, Sato M, Toba M, Masahashi W, Hashimoto-Gotoh T: **High-copy-number and low-copy-number plasmid vectors for lacZ alpha-complementation and chloramphenicol- or kanamycin-resistance selection**. *Gene* 1987, 61(1): 63-74.
5. Schafer A, Tauch A, Jager W, Kalinowski J, Thierbach G, Puhler A: **Small mobilizable multi-purpose cloning vectors derived from the Escherichia coli plasmids pK18 and pK19: selection of defined deletions in the chromosome of Corynebacterium glutamicum**. *Gene* 1994, 145(1): 69-73.
6. Dennis JJ, Zylstra GJ: **Plasposons: modular self-cloning minitransposon derivatives for rapid genetic analysis of gram-negative bacterial genomes.** *Appl Environ Microbiol* 1998, 64(7): 2710-2715.
