# Supplementary material for: Ralstonia solanacearum fatty acid composition is determined by interaction of two 3-ketoacyl-acyl carrier protein reductases encoded on separate replicons
Source: BMC Microbiol. 2015 Oct 22;15:223. doi: 10.1186/s12866-015-0554-x (PMC4618531; doi:10.1186/s12866-015-0554-x)
Supplement: Additional file 5: Table S3. — Sequences of the RT-PCR primers used in this work. [file 12866_2015_554_MOESM5_ESM.docx]

**Table S3. Sequences of the RT-PCR primers used in this work**

| **Primer name** | **Primer sequence (5’ to 3’)** |
| --- | --- |
| **RSp1020-F** | CGACAACGCGTCGGTACC |
| **RSp1020-R** | ACCACGATCGACAGGATG |
| **RSp1014-F** | GATCTTCGTGATCCTGCT |
| **RSp1014-R** | GCAGCAGTAGGCCTGCTG |
| **RSp0852-F** | GTACCGAGGCATTCAGTC |
| **RSp0852-R** | GAGCAGATCGTCGACGCC |
| **RSp0873-F** | TATCAGCTCGTCGACCAC |
| **RSp0873-R** | GCCTGCCAGCCGGTATTG |
| **RSp0875-F** | ATGCTCGCGTGTTCAGAG |
| **RSp0875-R** | CAATTGGACACTGTCGCC |
| **RSp0877-F** | TGCAAGACCTGATCAAGC |
| **RSp0877-R** | GGACCCTGGCTCTTGCTC |
| **RSp0872-F** | ACCAACGACCAGATGCTG |
| **RSp0872-R** | GGAGACGTTGTCGATGAG |
| **RSp0865-F** | GCGATGCTGGTAGCGCTG |
| **RSp0865-R** | CAGCGCATTCAGGCACAG |
| **RSp0858-F** | ACGAGCTCCAGTACCAGG |
| **RSp0858-R** | CTCCTCCATGATCATCAG |
| **RSp0856-F** | ATCATCGAGACCTTCAAC |
| **RSp0856-R** | AACCTTGTGCAAGCGTAC |
| **RSp0855-F** | AACACGACGAACACGACG |
| **RSp0855-R** | ATCAGCTTGGCGAGCATC |
| **RSp0382-F** | GGCATCCAGCAGCCTGAC |
| **RSp0382-R** | TTGGTATTCCTTGTCCAG |
| **RSp0378-F** | CCCACGCTGGAGATCATC |
| **RSp0378-R** | AGCGGCTTGACGTGCACC |
| **RSc2971-F** | CAAGCAATGCCGTCGAAC |
| **RSc2971-R** | GCCATATTGATAGTTGGC |
| **RSc2682-F** | ATGGTGTACGACATCATG |
| **RSc2682-R** | CAGGTCTTCCAGCGTCAG |
| **RSp0880-F** | GCTGTCTGCAAGACGCTG |
| **RSp0880-R** | GACTTGAGCTTGAGCGAG |
| **RSc1756-F** | GGTCGGCTCGACCTGCTC |
| **RSc1756-R** | AGTTTCGTCCCGTTCATG |
| **RSp0162-F** | CACCATCGCCAAGGACAG |
| **RSp0162-R** | CACCTGGCATACCTTGGC |
| **rplM-F** | CCGCGAAGCCCCATGAG |
| **rplM-R** | TGTCCGTCGCGTCAATCA |
